# Early detection of clinically significant prostate cancer at diagnosis: a prospective study using a novel panel of *TMPRSS2:ETS* fusion gene markers

**DOI:** 10.1002/cam4.49

**Published:** 2013-02-03

**Authors:** Sam W. Chan, Phuong-Nam Nguyen, Philippe Violette, Fadi Brimo, Yosh Taguchi, Armen Aprikian, Junjian Z. Chen

**Affiliations:** 1 Division of Urology, Department of Surgery, Research Institute of the McGill University Health CenterMontreal, Quebec, Canada, H3G 1A4; 2 Department of Pathology, Research Institute of the McGill University Health CenterMontreal, Quebec, Canada, H3G 1A4

**Keywords:** Biopsy cohort, diagnosis and prognosis, ETS fusions, prostate cancer, *TMPRSS2*, urine test

## Abstract

We explore noninvasive clinical applications of multiple disease-specific fusion markers recently discovered in prostate cancer to predict the risk of cancer occurrence and aggressiveness at diagnosis. A total of 92 men who were prostate-specific antigen (PSA) screened and scheduled for diagnostic biopsy were enrolled for this study. Prospectively collected urine was blind coded for laboratory tests. RNA from urine sediments was analyzed using a panel of 6 *TMPRSS2:ETS* fusion markers with a sensitive quantitative PCR platform. The pathology reported 39 biopsy-positive cases from 92 patients (42.4%). In urine test, 10 unique combinations of fusion types were detected in 32 of 92 (34.8%) prebiopsy samples. A novel combination of fusion markers, termed Fx (III, IV, ETS), was identified with a sensitivity of 51.3% and an odds ratio of 10.1 in detecting cancer on biopsy. Incorporating a categorical variable of Fx (III, IV, ETS) with urine *PCA3* and serum PSA, a regression model was developed to predict biopsy outcomes with an overall accuracy of 77%. Moreover, the overexpression of Fx (III, IV, or ETS) was shown to be an independent predictor to the high-grade cancer, with a predictive accuracy of 80% when coupled with PSA density. The individualized risk scores further stratified a high-risk group that is composed of 92% high-grade cancers and a low-risk group that harbors mainly clinically insignificant cancers. In conclusion, we have identified a novel combination of fusion types very specific to the clinically significant prostate cancer and developed effective regression models to predict biopsy outcomes and aggressive cancers at diagnosis.

## Introduction

The serum prostate-specific antigen (PSA) test coupled with needle biopsy is the standard clinical practice in prostate cancer diagnosis, but it is limited by the outcomes of excessive negative biopsies, overdiagnosis of clinically insignificant cancers and significant false-negative biopsy rate [1–4]. There is currently no reliable method for early detection of the life-threatening form of cancer. As a seminal discovery [5], many prostate tumors contain a specific genetic change that involves the fusion of an androgen-regulated gene with an oncogene. The most common fusion involves androgen-regulated *TMPRSS2* gene with an oncogenic ETS family transcription factor *ERG* gene, which is reported in 50% of surgical prostate tumors [5–12]. Moreover, diverse *TMPRSS2:ERG* fusion subtypes have been uncovered, ranging from chromosomal rearrangements to fusion transcripts [10, 12–15]. This fusion gene and its many subtypes not only allow stratification of clinically aggressive forms of cancer [10,13], but also provide redundant and cancer-specific transcript markers for noninvasive cancer detection in bodily fluids. Indeed, the *TMPRSS2:ERG* fusion RNA is shown to be detectable in the urine of men with prostate cancer [16–18]. A common subtype of this fusion, in combination with urine *PCA3*, enhanced the predictive performance of serum PSA for prostate cancer risk and clinically relevant cancer on biopsy in a recent clinical study [19]. However, the single subtype-based test excludes multiple alternative fusion markers for informed clinical tests.

In addition to the common fusion with *ERG* gene, *TMPRSS2* is also fused to several other ETS transcription factor genes, such as *ETV1*,* ETV4*, and *ETV5*, in approximately 10% of prostate tumors [5–8, 20]. Intriguingly, many of the low-prevalent *TMPRSS2:ETS* fusion events are associated with aggressive and metastatic cancers [21,22]. Moreover, ETS genes have also been found to fuse with an increasing number of androgen-regulated genes, all at low prevalence. Among these genes, *ETV1* emerges as a highly connected ETS gene, fused to more than 10 different androgen-regulated genes [22–25]. These fusion genes along with others have expanded into an interconnected fusion network, consisting of a dominant fusion and many low-prevalent fusion genes that may or may not be mutually exclusive in clinical tumors. Together, these fusion events provide not only a common mechanism for androgen-regulated overexpression of ETS transcription factor genes, but also novel molecular markers that are only detectable in prostate cancer [26–28]. However, the clinical significance of the increasing number of novel fusion genes is poorly studied due to their low prevalence as individual events and the lack of effective tools. Therefore, the potential clinical application of low-prevalent fusion gene markers in prostate cancer detection has yet to be reported. As multiple genomic alterations in prostate tumors have been recently shown to define a group of patients with high-risk cancer [29], a panel of multiple fusion gene markers may provide a new perspective for urine-based detection of the genetic instability or heterogeneity, and for better stratification of the clinically significant prostate cancer.

We are among the first to use a panel of *TMPRSS2:ERG* subtype markers for urine-based prostate cancer detection with high specificity and sensitivity [30]. In this study, we hypothesize that multiple *TMPRSS2:ERG* fusion subtypes and additional low-prevalent *TMPRSS2:ETS* fusion genes are collectively more informative than any single marker alone in the noninvasive detection and stratification of clinically significant prostate cancer. To test this hypothesis, a new panel of *TMPRSS2:ETS* fusion gene markers were investigated in the prospectively collected urine from PSA-screened men scheduled for diagnostic biopsy. We demonstrated for the first time, the feasibility of incorporating fusion subtypes and low-prevalent fusion genes into clinical practice. Moreover, we identified several alternative fusion markers very specific to clinically significant cancers and developed effective regression models to predict the risk of both cancer occurrence and aggressiveness prospectively.

## Materials and Methods

### Human subjects

Patients who were screened by serum PSA and scheduled for diagnostic needle biopsy were recruited at the prostate cancer clinics at the McGill University Health Center (MUHC). The research protocol was approved by the MUHC institutional review board and written informed consent was obtained from every participant. A total of 97 patients were recruited from April to November 2010 to form a prebiopsy cohort, among which 92 patients generated informative samples for molecular analysis (Fig. S1). Urine was collected post attentive digital rectal exam (DRE) and prior to needle biopsy. The prebiopsy urine specimens were coded for anonymity. Laboratory investigators were blinded to sample allocation for prospective molecular diagnosis using a panel of molecular markers. The results from this panel were then compared to needle biopsy results in a double-blinded protocol. The pathology of each biopsy with 10 needle cores was reviewed by a single genitor-urinary pathologist at MUHC. All included slides were assigned a grade according to the modified Gleason grading system. Men with previous treatment for prostate cancer or with repeat biopsy were excluded from this study. The baseline clinical–pathological information of the study cohort and 1-year follow-up are provided in Table 1.

**Table 1:** Baseline clinical and pathological characteristics of biopsy cases

Characteristic	Biopsy positive (39)	Biopsy negative (53)
Age		
Mean^1^	67.46 ± 7.85	68.55 ± 8.12
Median^2^	68 (50–86)	70 (52–87)
*n*	39	53
Pre-Bx PSA (ng/mL)		
Mean	15.67 ± 42.99	6.75 ± 4.07
Median	6.71 (1.18–271.57)	5.87 (0.74–25.25)
*n*	39	53
F/U PSA (ng/mL)		
Mean	31.29 ± 99.42	7.55 ± 5.65
Median	8.26 (1.19–484.89)	6.35 (9–145)
*n*	23	23
Prostate size (cc)		
Mean	40.46 ± 19.93	48.73 ± 23.65
Median	35.00 (16–110)*	46 (3–60)
*n*	39	51
PSA density		
Mean	0.37 ± 0.71	0.17 ± 0.14
Median	0.2 (0.05–4.24)**	0.12 (0.03–0.63)
*n*	39	51
Gleason score		
Mean	6.74 ± 1.02	NA
Median	6 (6–9)	NA
*n*	39	53
HG PIN^3^		
*n*	17	16
No. of cores positive		
Mean	3.90 ± 2.99	NA
Median	3 (1–10)	NA
*n*	39	53
Maximum percentage cancer involvement in any core		
Mean	44.23 ± 33.77	NA
Median	40 (5–100)	NA
*n*	39	53
Treatment		
RP^4^ (*n*)	9	0

PIN, prostatic intraepithelial neoplasia; PSA, prostate-specific antigen.

1 Mean with standard deviation, Student's *t*-test (*P <**0.05, **P <**0.01).

2 Median with min–max values, Mann–Whitney test (*P <**0.05, **P <**0.01).

3 High-grade PIN.

4 Radical prostatectomy.

### Urine collection and whole-transcriptome cDNA library preparation

From each subject, 10–40 mL of the first voided urine post DRE was collected in a sterile collection cup containing RNA/DNA preservatives (Sierra Molecular, Incline Village, NV) and processed within 4 h of sampling. Urine sediments were collected by low-speed centrifugation at 4°C and resuspended in TRIzol Reagent (Invitrogen, Carlsbad, CA) for immediate RNA extraction or stored at −80°C until use. Total RNA was extracted from urine sediments using a miRNAeasy Mini kit (Qiagen, Germantown, MD) according to the manufacturer's instructions with minor modifications. A whole-transcriptome cDNA library was generated for each sample using a TransPlex WTA2 kit (Sigma-Aldrich, St. Louis, MO) [30].

### Detection of multiple molecular markers

Real-time quantitative PCR (qPCR) was used to detect a panel of fusion markers consisting of three *TMPRSS2:ERG* fusion subtypes (I, III, and IV) and three *TMPRSS2:ETS* fusion genes (*ETV1*,* ETV4*, and *ETV5*) using the previously established protocol [30]. Additional molecular markers were also quantified, including two *ERG* markers targeting exons 5–6 and 6–7, *PCA3*,* PSA*, and the housekeeping gene *GAPDH*. The probe sequences, amplification features, and gene locations are listed in Table S1 and Figure S2. Briefly, 9 ng of cDNA was amplified in a 20-μL reaction containing 1× SYBR Green Supermix (Bio-Rad, Hercules, CA) and 300 nmol/L of each forward and reverse primers, using a two-step amplification program. The qPCR program consisted of initial denaturing at 95°C for 1.5 min, followed by 50 cycles of a two-step reaction at 95°C for 15 sec, and 67–70°C (varying for marker pairs) for 30 sec. The qPCR was performed using the MyiQ real-time PCR system (Bio-Rad). The relative expression of each target gene was normalized to *GADPH* for nonfusion markers, or both *GAPDH* and *PSA* for fusion markers using the comparative *C*_t_ method (Applied Biosystems User Bulletin 2, Foster City, CA). A control sample was included in all amplifications to serve as a common calibrator for relative expression.

### Data analysis

All statistical analyses except for the DeLong's test were performed using IBM SPSS statistics 19, version 19.0.0 (IBM Corporation, Armonk, NY). The DeLong's test was performed using R, version 2.13.1 (R Project for Statistical Computing, http://www.R-project.org). Two-sided tests were used for all comparisons and *P* values <0.05 were considered statistically significant. The REMARK guidelines were followed in data analysis [31].

Associations between biopsy outcomes, molecular subgroups, and clinical–pathological variables were assessed with Student's *t*-test (parametric), Mann–Whitney test (nonparametric), Fisher's exact test or chi-square test (categorical), or Spearman's ρ. Diagnostic values of biomarkers were quantified with sensitivity, specificity, predictive accuracy, odds ratio, and area under the curve (AUC) in the receiver operating characteristic (ROC) curve. The significance in AUCs between different markers was examined with the DeLong's test [32]. The probability of different combination of biomarkers to predict risk of cancer occurrence (i.e., biopsy outcome) or risk of high-grade cancer (i.e., Gleason score ≥7) was assessed using multivariate logistic regression models.

For cancer risk assessment, individual molecular scores were computed from regression models for each subject and used directly to stratify men into three risk groups (i.e., high, intermediate, and low risk) for either biopsy outcome in all biopsy cases or high-grade cancer in biopsy-positive cases. The relative risk (RR) between different risk groups was calculated to measure the probability of biopsy outcome, high-grade cancer, high risk of recurrence (Gleason ≥8, pre-bx PSA ≥ 20 ng/mL or stage T3a) defined by the National Comprehensive Cancer Network (NCCN) guidelines (http://www.nccn.org), features indicative of clinical significance (i.e., prostate-specific antigen density [PSAD] ≥ 0.15 ng/mL or positive cores ≥3 or maximum cancer involvement in a single core ≥50%), and clinically insignificant cancers (i.e., Gleason ≤6, 1–2 positive cores, <50% cancer involvement in any core and PSAD < 0.15 ng/mL) defined by the Epstein criteria [33,34].

## Results

### Fusion type and clinical sensitivity of a panel of TMPRSS2:ETS fusion markers

The common fusion transcript between TMPRSS2-exon1 and ERG-exon4 (TMP-e1:ERG-e4), subtype I in this study, was the primary marker used in urine-based detection of prostate cancer [16–19]. We previously reported a panel of *TMPRSS2:ERG* fusion-subtype markers for urine-based cancer detection with high specificity and sensitivity [30]. In this study, we expanded a new panel of *TMPRSS2:ETS* fusion markers (Table S1 and Fig. S2) and demonstrated reproducible detection of individual fusion markers in a dynamic range from 1.8 million to 18 copies using the qPCR platform (Fig. S3). Using this expanded fusion panel in a urine test, we detected 10 unique combinations of fusion genes and/or subtypes that we termed as “fusion types,” distributed with various frequencies in 32 cases (34.8%) from a prebiopsy cohort of 92 patients (Table 2). Six of the fusion types contained the common subtype I. The remaining four fusion types contained no subtype I and were detected in five of 32 (15.6%) fusion-positive cases. Significantly, we showed for the first time the detection of three low-prevalent *TMPRSS2:ETS* fusion genes in four urine specimens, among which two of the specimens also had at least one *TMPRSS2:ERG* subtype. The pathology, on the other hand, identified 39/92 (42.4%) biopsy-positive cases (Table 1). We reasoned that the diverse fusion types provided not only redundant fusion markers for improved sensitivity in cancer detection but also a molecular basis for stratification of cancer risks. Indeed, we demonstrated that different fusion types exhibited different predictive values on biopsy outcomes; the most informative fusion markers composed of *TMPRSS2:ERG* subtype III, IV or any *TMPRSS2:ETS* (*ETV1*,* ETV4*,* ETV5*) fusions, a special combination we termed Fx (III, IV, ETS). When used as a categorical variable, this combination of fusion markers had a sensitivity of 51.3% and a specificity of 90.6% with an odds ratio of 10.1 in detecting prostate cancer on biopsy (Table 3). Significantly, this novel combination of fusion markers outperformed fusion types containing the common subtype I in prospective cancer detection. Indeed, six of seven cases detected with the common subtype alone were biopsy negative. Taken together, fusion-typing of a novel panel of *TMPRSS2:ETS* fusion markers allows detection of diverse fusion types in urine; multiple alternative fusion markers other than the common subtype I provide improved detection of prostate cancer in prebiopsy patients. To rule out potential contamination in fusion detection, fusion-positive results were independently validated in a second aliquot of original RNA from all 32 fusion-positive cases (Fig. S1 and Table S2), in corresponding prostatectomy cancer tissues from three available fusion-positive cases (Table S3) and by DNA sequencing (Fig. S4).

**Table 2:** Fusion types: combination of fusion genes and subtypes found in individual biopsy patients

No. of patients (*n*)	TMP:ERG I (T-e1:E-e4)	TMP:ERG III (T-e1:E-e2)	TMP:ERG IV (T-e1:E-e5)	TMP:ETV1 (T-e1:V1-e6)	TMP:ETV4 (T-e1:V4-e5)	TMP:ETV5 (T-e1:V5-e2)	Fusion types^1^
7	+						TMP:ERG I
7	+	+					TMP:ERG (I + III)
4	+		+				TMP:ERG (I + IV)
1	+					+	TMP:ERG I + TMP:ETV5
2		+					TMP:ERG III
1			+				TMP:ERG IV
7	+	+	+				TMP:ERG (I + III + IV)
1	+		+	+			TMP:ERG (I + IV) + TMP:ETV1
1					+		TMP:ETV4
1						+	TMP:ETV5

1 Combination of fusion markers detected in the same patient.

**Table 3:** Sensitivity and specificity of models based on different *TMPRSS2:ETS* fusion types to predict biopsy outcome

Features	Pre-bx PSA^1^	TMP:ERG I^2^	Fx (III, IV, ETS)^3^	All fusions^4^
Frequency (%)	–	27/92 (29.35)	25/92 (27.17)	32/92 (34.78)
Sensitivity (%)	9/30 (23.1)	17/39 (43.6)	20/39 (51.3)	21/39 (53.8)
Specificity (%)	45/53 (84.9)	43/53 (81.1)	48/53 (90.6)	42/53 (79.2)
Overall percentage	58.7	65.2	73.9	68.5
Odds ratio (OR) (95% CI)	1.629 (0.960–2.763)	3.323 (1.305–8.463)	10.105 (3.315–30.808)	4.455 (1.784–11.121)
*P*	0.070	0.012	<0.001	0.001

PSA, prostate-specific antigen.

1 Prebiopsy serum PSA (quantitative variable).

2 Any fusion-type event containing TMP:ERG subtype I fusion (categorical variable).

3 Any fusion-type event containing TMP:ERG subtype III or IV or other TMP:ETS (*ETV1*,* ETV4*,* ETV5*) (categorical variable).

4 Any fusion-type event containing TMP:ERG subtype I or III or IV or other TMP:ETS (*ETV1, ETV4*,* ETV5*) (categorical variable).

### Distinct molecular subgroups stratified by biopsy outcome and fusion status

**Figure 1:**
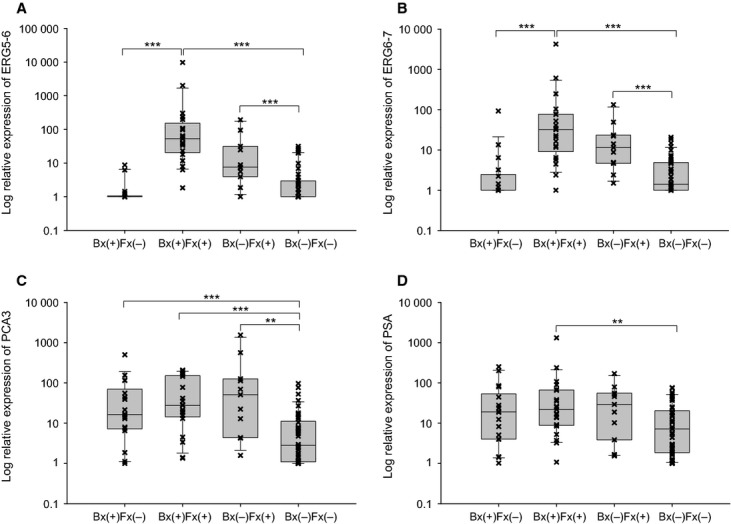
Distinctive molecular subgroups stratified by biopsy outcomes and fusion status in urine. A total of 92 biopsy patients were stratified into the four subgroups based on biopsy outcomes (Bx) and fusion status (Fx): Bx(+)/Fx(−) (*n* = 18), Bx(+)/Fx(+) (*n* = 21), Bx(−)/Fx(+) (*n* = 11), and Bx(−)/Fx(−) (*n* = 42). (A, B), the expression levels of two *ERG* markers (ERG [5–6] and [6–7], respectively) in urine were stratified by Bx and Fx status. (C, D), the expression levels of *PCA3* and *PSA* in urine were stratified by Bx and Fx status. The relative expression was calculated using ∆∆ threshold cycle method and transformed into log(*x* + 1) values. Mann–Whitney test was performed (**P < 0.01, and ***P < 0.001).

Biopsy outcomes stratified by fusion status (all fusion types) identified four distinctive molecular subgroups exhibiting different molecular (Fig. 1) and clinical–pathological (Table 4) features. For example, a biopsy-positive/fusion-positive [Bx(+)/Fx(+)] subgroup exhibited a very significant increase in urine expression of cancer-specific markers *ERG*(*5*-*6*), *ERG*(*6-7*), and *PCA3* when compared with a double-negative subgroup [Bx(−)/Fx(−)]. This result was consistent with our previous observation for a strong correlation between *TMPRSS2:ERG* fusion and *ERG* expression levels in urine of prostate cancer patients [30]. In contrast, a biopsy-positive/fusion-negative subgroup [Bx(+)/Fx(−)] exhibited a residual level of *ERG* markers, but a significantly elevated *PCA3* expression. Thus, the *PCA3* marker may be informative to identify prostate cancer in the Bx(+)/Fx(−) subgroup. It is useful to note that the overall urine *PSA* level was similar between [Bx(+)/Fx(+)] and [Bx(+)/Fx(−)] subgroups (Fig. 1D). Thus, the Fx(−) status in Bx(+) cases could not simply be attributed to the biased sampling. On the other hand, a small subgroup of biopsy-negative/fusion-positive [Bx(−)/Fx(+)] cases (*n* = 11) was also identified in this study, which had a molecular profile similar to the Bx(+)/Fx(+) subgroup. Interestingly, most cases detected with the common subtype I alone belonged to this subgroup. However, the Bx(−)/Fx(+) subgroup was shown to be associated with older age (*P* < 0.001) and increased incidence of high-grade prostatic intraepithelial neoplasia (PIN) lesions (*P* < 0.05) as compared with the double-negative subgroup (Table 4). These features raise the possibility that the Bx(−)/Fx(+) subgroup has an elevated risk for either “false” biopsy-negative events or future development of cancer in at least some of the cases (e.g., cases with both fusion-positive and with HG PIN lesions). Finally, the double-negative subgroup [Bx(−)/Fx(−)] was shown to exhibit baseline levels of molecular and clinical features as compared with the other three subgroups.

**Table 4:** Clinical and pathological characteristics of four distinct molecular subgroups stratified by biopsy (Bx) and fusion (Fx) status

Clinical parameters^1^	Bx(+)/Fx(−)	Bx(+)/Fx(+)	Bx(−)/Fx(+)	Bx(−)/Fx(−)
No. of cases (*n*)	18	21	11	42
Age	69.50 (54–86)	68 (50–81)	75.00 (65–83)***	66 (52–87)
Pre-Bx prostate-specific antigen (ng/mL)	6.60 (2.33–271.57)	6.71 (1.18–54.46)	7.51 (1.29–25.25)	5.83 (0.74–14.31)
Prostate Size (cc)	35.00 (19–110)	35 (16–88)	46 (19–80)	46.50 (9–145)
Prostate-specific antigen density	0.21 (0.05–4.24)**	0.18 (0.05–1.88)*	0.15 (0.05–0.63)	0.12 (0.03–0.56)
HG PIN (*n*)^2^	8	9	6*	10
HG PIN core no. >2 (*n*)^2^	4	7	4*	6
Gleason score	6 (6–9)	6 (6–9)	–	–
No. of cores with cancer	3 (1–10)	3 (1–10)	–	–
Max% inv. of single core	40 (5–100)	40 (5–100)	–	–
Treatment (*n*)^3^	4 RP^3^	5 RP	–	–

PIN, prostatic intraepithelial neoplasia.

1 Expressed as median with min–max values unless indicated differently, Mann–Whitney test (vs. Bx(−)/Fx(−) where applicable; *P <**0.05, **P <**0.01, ***P <**0.001).

2 Number of cases, chi-square test (*P *<**0.05).

3 Radical prostatectomy.

### Individualized risk model to predict cancer occurrence at prostate biopsy

**Figure 2:**
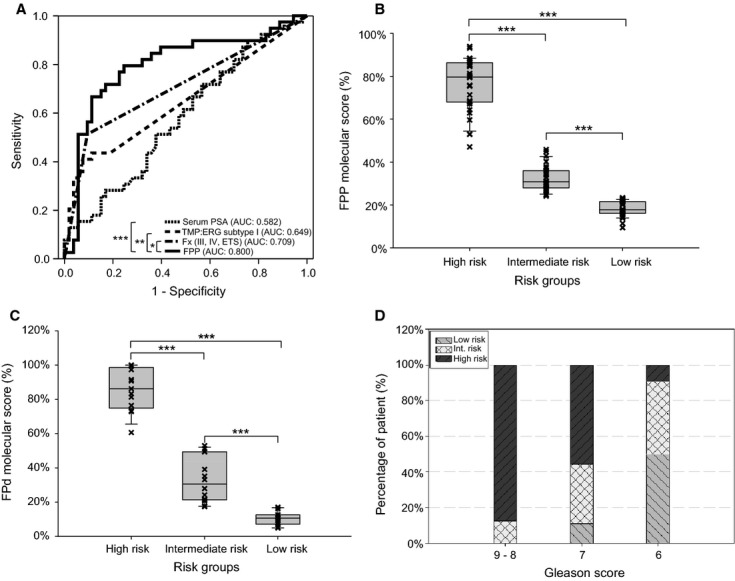
Molecular risk groups to predict either biopsy outcomes (A, B) or high-grade prostate cancers (C, D) in a prebiopsy cohort. (A) Receiver operating characteristic (ROC) curves for pre-Bx serum prostate-specific antigen (PSA) (blue), *TMPRSS2:ERG* (TMP:ERG) fusion subtype I (green), informative fusion types Fx (III, IV, ETS) (brown), and the FPP scores (purple). The FPP score [Fx (III, IV, ETS) + PCA3 + Pre-Bx PSA] was used for the prediction of biopsy outcomes in the prebiopsy cohort (*n* = 92). (B) The FPP scores were used to stratify prebiopsy patients into high- (*n* = 31), intermediate- (*n* = 30), and low (*n* = 31)-risk groups. (C) The FPd scores [Fx (III, IV, ETS) + prostate-specific antigen density] were used to stratify biopsy-positive patients (*n* = 39) into high- (*n* = 13), intermediate- (*n* = 14), and low (*n* = 12)-risk groups to high-grade cancers (Gleason >6). (D) Distribution of high-, intermediate-, and low-risk groups (%) in patients with total Gleason scores of 9–8 (*n* = 8), 7 (*n* = 9), and 6 (*n* = 22). The area under the curve of the ROC curves were compared with DeLong's test (*P <**0.05, **P <**0.01, and ***P <**0.001). The Mann–Whitney test was performed between each risk group (***P < 0.001).

The combined detectability of the informative fusion markers [i.e., Fx (III, IV, ETS)] had an improved predictive value on cancer occurrence. By incorporating the categorical variable of fusion types Fx (III, IV, ETS) with log-transformed continuous variables of *PCA3* and serum PSA, a logistic regression model was developed to calculate an individualized molecular score for optimal prediction of biopsy outcomes (Table 5). As demonstrated in the ROC curve analysis, the Fx (III, IV, ETS) + PCA3 + serum PSA (FPP) score had a significantly increased AUC (0.80) as compared with serum PSA (0.58, *P* < 0.001), *TMPRSS2:ERG* subtype I (0.65, *P* < 0.01), or Fx (III, IV, ETS) fusion type (0.71, *P* < 0.05) in the prebiopsy cohort of 92 patients (Fig. 2A). For clinical-friendly applications, the FPP molecular scores were used to stratify prebiopsy patients prospectively into three distinctive risk groups: the high-risk group (FPP scores [1–0.47], *n* = 31), the intermediate-risk group (scores [0.47–0.25], *n* = 30), and the low-risk group (scores [0.25–0], *n* = 31) (Fig. 2B). As such, the positive predictive value (PPV) for prostate cancer detection was 81% in the high-risk group, but only 30% and 16% in the intermediate- and low-risk groups (Table 6). On the other hand, a small fraction of biopsy-negative cases were identified in the high-risk group; such cases may be prioritized for repeat biopsies to identify potentially false-negative biopsies. Meanwhile, all except two biopsy-negative cases in the low-risk group were also negative to any fusion markers and could be truly low-risk subjects to prostate cancer. Interestingly, three Bx(−) patients from the low-risk group were biopsied again within 12 months and remained negative in the follow-up biopsy. However, the FPP model was not effective to predict significant cancers when only the biopsy-positive cases were considered (Table 6, but also Table S4).

**Table 5:** A logistic regression model based on informative fusion markers [Fx (III, IV, ETS)], *PCA3*, and prebiopsy serum PSA to predict cancer risk on biopsy

			Univariable logistic regression models			Multivariable logistic regression model^1^		
Biopsy cohort (*n*)	Dependent variable	Diagnostic Variable	OR (95% CI)	*P*	Overall accuracy (%)	OR (95% CI)	*P*	Overall accuracy (%)^2^
		Fx (III, IV, ETS)^3^	10.11 (3.32–30.81)	<0.0001	73.9	7.10 (2.20–22.89)	0.001	
92	Biopsy outcome	PCA3^4^	1.34 (1.11–1.60)	0.002	65.2	1.22 (1.00–1.49)	0.045	77.2^5^
		Serum PSA^4^	1.63 (0.96–2.76)	0.07	58.7	1.58 (0.89–2.77)	0.116	

PSA, prostate-specific antigen.

1 Hosmer–Lemeshow Goodness-of-Fit of logistic regression model: *P* = 0.307.

2 Defined as (true positives + true negatives)/all.

3 TMP:ERG subtype III or IV, or TMP:ETS (ETV 1, 4, or 5) (binary categorical variable).

4 PCA3 and serum PSA were log-transformed continuous variables.

5 61.5% sensitivity and 88.7% specificity at 50% cut-off value.

**Table 6:** Risk groups to cancer occurrence stratified by the FPP molecular scores

	High	Intermediate	Low
Risk score^1^			
* n* (%)	31 (34)	30 (33)	31 (34)
Median (min–max)	0.7967 (0.4707–0.9391)	0.3077 (0.2415–0.4582)	0.1772 (0.0943–0.2335)
Cancer			
*n* (%)^2^	25 (81)	9 (30)	5 (16)
RR (H vs. L), *P*^3^	5.000 (2.199–11.370), *P *<**0.0001		
RR (H vs. [I + L]), *P*^4^	3.514 (2.150–5.743), *P *<**0.0001		
Gleason score ≥7			
*n* (%)^5^	14 (56)	3 (33)	0 (0)
RR (H vs. L), *P*	Undefined, *P *=**0.0447		
RR (H vs. [I + L]), *P*	2.613 (0.9038–7.557), *P *=**0.0490		
High risk of recurrence by NCCN^6^			
*n* (%)^5^	7 (28)	2 (22)	0 (0)
RR (H vs. L), *P*	Undefined, *P *=**0.3041		
RR (H vs. [I + L]), *P*	1.960 (0.469–8.183), *P *=**0.4448		
No. of cores positive ≥3			
*n* (%)^5^	16 (64)	6 (67)	2 (40)
RR (H vs. L), *P*	1.600 (0.526–4.871), *P *=**0.3644		
RR (H vs. [I + L]), *P*	0.960 (0.584–1.577), *P *=**1		
Max.% cancer inv. in any core ≥50%			
*n* (%)^5^	12 (48)	2 (22)	0 (0)
RR (H vs. L), *P*	Undefined, *P *=**0.0657		
RR (H vs. [I + L]), *P*	3.360 (0.874–12.920), *P *=**0.0444		

NCCN, National Comprehensive Cancer Network; PSA, prostate-specific antigen; RR, relative risk.

1 FPP molecular score: FPP regression model = −2.890 + 1.960*Fx(III, IV, ETS) + 0.201*log 2 (PCA3 + 1) + 0.454*log 2 (serum PSA + 1), Risk score = 1/(1 + e^(−FPP)^), where e is the number of ∼2.71828.

2 Percentage of each risk group or positive predictive value in a total of 92 prebiopsy patients.

3 RR between high (H)- and low (L)-risk groups with 95% CI, *P* value of Fisher exact test.

4 RR between high- and intermediate + low (I + L)-risk groups with 95% CI, *P* value of Fisher exact test.

5 Percentage of each risk group or positive predictive value in 39 biopsy-positive patients.

6 Gleason score ≥8, PSA ≥20 ng/mL or T3a as defined by the NCCN guidelines.

### Individualized risk model to predict the risk of aggressive cancer

One of the most challenging tasks of early cancer detection is to identify the aggressive forms of cancer at diagnosis. We explored risk models to predict high-grade cancers (Gleason ≥7) in the context of biopsy-positive cases. Unlike the detectability (i.e., categorical variable) of Fx (III, IV, or ETS) used in predicting biopsy outcomes, the overexpression profiles of these fusion markers were more informative to identify clinically significant cancers. Using a common calibrating sample sparked with fusion molecules, we devised a strategy that ranked the relative quantity of different informative fusion markers as a new continuous variable. Instead of using each fusion marker separately with reduced statistic power, we proposed to calculate the quantitative feature of combined fusion markers based on the sum of quantity values (or the highest relative quantity) of fusion markers in each sample. Coupling this new continuous variable of Fx (III, IV, or ETS) with the continuous variable of PSA density (PSAD) that we termed as Fx (III, IV, ETS) + PSA density (FPd), a logistic regression model was developed to predict the risk of high-grade cancer (Gleason ≥7) with an overall accuracy of 80% (Table 7). The essentially same scores were obtained when the combined fusion markers were re-ranked based on the highest quantity in each sample (data not shown). The FPd scores were then used to stratify biopsy-positive cases equally into high-, intermediate-, and low-risk groups for clinically significant cancers (Fig. 2C and D). We demonstrated that the high-risk group not only identified 92.3% (12/13) Gleason ≥7 cancers (*P* < 0.0001) but also associated with the high-risk-of-recurrence cases (*P* < 0.001) as defined by the NCCN guidelines (Table 8). Indeed, the FPd molecular scores were highly correlated with Gleason scores (*r* = 0.65, *P* < 0.0001), the number of positive cores (*r* = 0.47, *P* < 0.01), and the percentage of cancer involvement in a single core (*r* = 0.61, *P* < 0.001) when analyzed by the Spearman's ρ (Table 9). Conversely, a low-risk group for cancer progression was also identified using the same score system, which consisted of 91.6% (11/12) cases with Gleason 6 cancers. Coincidentally, six clinically insignificant cancers that were identified among the 39 Bx(+) cases by the Epstein criteria all belonged to this low-risk group. The significant association (*P* < 0.01) of the low-risk cancers based on molecular scores with the insignificant cancers based on pathology may suggest an indolent property for such cancer patients. Indeed, three Bx(+) cases (Gleason 6) from the low-risk group exhibited no progression in 12-month follow-up biopsies, while two Bx(+) cases (Gleason 6) from the intermediate-risk group failed to detected cancer cells in repeat biopsy (data not shown). However, long-term follow-up is required to determine the clinical outcomes that are beyond the scope of this study.

**Table 7:** A logistic regression model based on informative fusion types [Fx (III, IV, ETS)] and PSAD to predict risk of aggressive cancer on biopsy

			Univariable logistic regression models			Multivariable logistic regression model^1^		
Biopsy cohort (*n*)	Dependent variable	Diagnostic variable	OR (95% CI)	*P*	Overall accuracy (%)	OR (95% CI)	*P*	Overall accuracy (%)^2^
39	Gleason score (≥7 or 6)	Fx (III, IV, ETS)^3^	2.06 (1.11–3.81)	0.022	69.2	2.23 (1.09–4.59)	0.029	79.5^4^
		PSAD^5^	21942.60 (7.80–6.18 × 10^7^)	0.014	71.8	51723.691 (11.54–2.31 × 10^8^)	0.011	

PSAD, prostate-specific antigen density.

1 Hosmer–Lemeshow Goodness-of-Fit of logistic regression model: *P* = 0.910.

2 Defined as (true positives + true negatives)/all.

3 TMP:ERG subtype III, IV, or TMP:ETS (ETV 1, 4, or 5) (log-transformed continuous variable).

4 70.6% sensitivity and 86.4% specificity at 50% cut-off value.

5 PSAD continuous variable.

**Table 8:** Risk groups to clinically significant cancers stratified by the FPd molecular scores

	High	Intermediate	Low
Risk score^1^			
*n* (%)	13 (33)	14 (36)	12 (31)
Median (min–max)	(0.6064–1.0000)	(0.1735–0.5297)	(0.0489–0.1706)
Gleason score ≥7			
*n* (%)^2^	12 (92)	4 (29)	1 (8)
RR (H vs. L), *P*^3^	10.150 (1.556–66.260), *P* < 0.0001		
RR (H vs. [I + L]), *P*^4^	4.800 (2.149–10.720), *P* < 0.0001		
High risk of recurrence by NCCN^5^			
*n* (%)^2^	8 (62)	1 (7)	0 (0)
RR (H vs. L), *P*	Undefined, *P* = 0.0016		
RR (H vs. [I + L]), *P*	16.000 (2.232–114.700), *P* = 0.0002		
No. of cores ≥3			
*n* (%)^2^	11 (85)	8 (57)	5 (42)
RR (H vs. L), *P*	2.031 (1.000–4.125), *P* = 0.0414		
RR (H vs. [I + L]), *P*	1.692 (1.080–2.651), *P* = 0.0449		
Max.% cancer inv. in any core ≥50%			
*n* (%)^2^	9 (69)	5 (36)	0 (0)
RR (H vs. L), *P*	Undefined, *P* = 0.0005		
RR (H vs. [I + L]), *P*	3.600 (1.512–8.570), *P* = 0.0041		
Insignificant cancer by the Epstein criteria^6^			
*n* (%)^2^	0 (0)	0 (0)	6 (50)
RR (L vs. H), *P*	Undefined, *P* = 0.0052		
RR (L vs. [H + I]), *P*	Undefined, *P* = 0.0003		

NCCN, National Comprehensive Cancer Network; PSAD, prostate-specific antigen density; RR, relative risk.

1 FPd molecular score: FPd = −3.481 + 0.803*log 2 Fx(III, IV, ETS) + 10.854*PSAD, High-grade cancer risk score = 1/(1 + e^(−FPd)^).

2 % Of each risk group or positive predictive value in 39 biopsy positive patients.

3 RR between high- and low-risk group with 95% CI, *P* value of Fisher exact test.

4 RR between high- and intermediate + low-risk group with 95% CI, *P* value of Fisher exact test.

5 Gleason score ≥8, PSA ≥ 20 ng/mL or T3a as defined by the NCCN guidelines.

6 Gleason score ≤6, no. of cores ≤2, percentage cancer involvement ≤50%, and PSAD ≤ 0.15 ng/mL met at the same time. RR between low- and high-, low- and high + intermediate (H + I)-risk group with 95% CI, *P* value of Fisher exact test.

**Table 9:** Correlation of clinical–pathological and molecular characteristics with the FPd molecular scores for aggressiveness

Parameter	No. of patients (*n*)	FPd score^1^ (Spearman *r*_s_)	*P*
Age	39	0.167	0.311
Prebiopsy PSA	39	0.536	<0.0001
Prostate size	39	−0.467	0.003
PSA density	39	0.845	<0.0001
Gleason score	39	0.659	<0.001
No. of cores with cancer	39	0.423	0.007
Max. % cancer inv. in any core	39	0.564	<0.0001
Urine *PSA*	39	0.185	0.258
Urine *PCA3*	39	0.083	0.616
Urine *ERG*(*5-6*)	39	0.227	0.164

PSA, prostate-specific antigen.

1 Spearman's rank correlation test to measure correlation of the FPd score with each parameter.

## Discussion

In this study, we report a prospective study for molecular diagnosis of PSA-screened patients who are scheduled for diagnostic biopsy (prebiopsy cohort) using a novel panel of both common *TMPRSS2:ERG* subtypes and low-prevalent *TMPRSS2:ETS* (*ETV1*,* ETV4*, and *ETV5*) fusion markers. We demonstrated for the first time the clinical utility of multiple low-prevalent fusion markers and diverse fusion types in urine-based cancer detection. Moreover, we identified multiple alternative fusion genes and subtypes very specific to clinically significant prostate cancers than the common subtype I, and developed effective regression models to predict both the risk of cancer occurrence and the risk of aggressive cancer prospectively.

Prostate cancer is characterized by its extensive clinical heterogeneity; early stratification of aggressive disease from a majority of indolent cancers at diagnosis is a critical clinical task in cancer management and treatment. Our work builds on an increasing number of disease-specific fusion genes recently discovered in prostate cancer [26–28]. We argue that a panel of multiple fusion markers is superior to any single one alone in the noninvasive detection and stratification of clinically significant tumors. First, both high- and low-prevalent fusion genes/subtypes together provide improved clinical sensitivity through redundant transcript markers and mutually exclusive nature of some fusion genes [35]. Second, the panel approach offers rich genetic diversity for better stratification of heterogeneous tumors through unique genetic and molecular profiles associated with each individual patient. Some fusion subtypes are shown to have prognostic values [10], while many low-prevalent fusion events are associated with aggressive cancers [21,22]. Third, the panel approach provides new insights into the complexity and the extent of genetic heterogeneity or instability associated with multiple independent fusion events detectable in the same patients [35]. Such information may be very useful for noninvasive identification of a group of highly aggressive prostate cancer defined by extensive genomic alterations [29]. To explore this new perspective, we have developed a qPCR platform for urine-based detection of multiple fusion markers. This platform is shown to have a high specificity (>99%) in a negative control group and a sensitivity of detecting a single fusion-positive cancer cell in at least 3000 normal cells in men's urine sediments [30]. Reproducible detection of fusion-subtype molecules are also demonstrated in the dynamic range from 1.8 million to 18 copies (Fig. S3) and from independent preparation of the same samples (Fig. S1). Using this sensitive platform to screen a panel of six *TMPRSS2:ETS* fusion markers, we not only detected diverse fusion types in urine of prebiopsy patients but also identified several alternative fusion markers very specific to biopsy outcomes. The informative fusion markers, Fx (III, IV, or ETS), are collectively more sensitive than the dominant fusion subtype I in prospective prostate cancer detection. Moreover, by incorporating the categorical variable of Fx (III, IV, or ETS) with urine *PCA3* and serum PSA, the multivariate FPP model not only has an overall predictive accuracy of 77% to overall prostate cancer detection, but also allows individualized stratification of prebiopsy patients into distinctive risk groups. As such, the PPV on the biopsy outcome is 81% in a high-risk group, but only 16% in a low-risk group. On the other hand, the combined quantity of Fx (III, IV, or ETS) in urine are shown to be an independent predictor to the high-grade cancers (*P* < 0.022). When coupled with PSAD, the FPd model detects the aggressive prostate cancer with an overall predictive accuracy of 80% in biopsy-positive patients. The resulting FPd scores further stratify not only a high-risk group that is composed of 92% high-grade cancer, but also a low-risk group that harbors mainly clinically insignificant cancers. However, it remains to be elucidated on the basis of different clinical implications between a single versus a panel of markers as well as between detectability and overexpression among the same set of informative fusion markers. Interestingly, the less common subtypes (III and IV) are frequently accompanied by the common subtype I in Bx(+) cases, but the common subtype alone is overrepresented in the [Bx(−)/Fx(+)] subgroup. Thus, the genetic complexity of fusion types and the active expression of less common fusion markers in urine may be associated with an active state of cancer proliferation and hence serve as better predictive markers to the biopsy outcomes and the risk of aggressive cancer. This hypothesis is further supported by the identification of multiple novel fusion subtypes associated with the high-risk cancers when the same urine specimens were screened by a DNA-chip-based high-throughput method (P.-N Nguyen and J. Z. Chen, unpubl. data). Therefore, the panel approach is better suited to identify clinical significant cancers over a single marker-based test. It is feasible to further improve the clinical sensitivity by including additional fusion markers (e.g., *SLC45A3*,* KLK2*, and so on) into the same platform, and to simplify the multimarker analyses into a single test using a qPCR-array format. However, it is necessary to point out that the assay based the new fusion panel was a laboratory-developed test and that our prospective study is limited by a relatively small sample size and short follow-up time. Independent validation studies are necessary and current ongoing to address these limitations by the authors.

In conclusion, we propose a urine-based clinical test for early detection of clinically significant prostate cancer using a panel of multiple *TMPRSS2:ETS* fusion markers. The extensive diversity of fusion types identified in urine of PSA-screened men provides better genetic and molecular bases to stratify the clinical heterogeneity of prostate cancer. Clinically applicable risk models are developed to generate individualized molecular scores to identify distinctive risk groups. As such, patients identified with a high molecular score will likely lead to the detection of aggressive forms of cancer in biopsy-positive cases or “false” diagnosis in biopsy-negative cases who should be prioritized for repeat biopsies. On the other hand, patients characterized with a low-risk score may represent indolent cancer in biopsy-positive cases or truly low-risk subjects in biopsy-negative cases. The former may be benefitted by conservative management such as active surveillance, while the latter may be excluded from further screening to reduce the biopsy burden. It is logical to expect that the conceptual advances and simultaneous analysis of all existing fusion markers using high-throughput technologies will provide a new paradigm for personalized molecular diagnosis of the clinically significant prostate cancer.

## Supporting Information

Figure S1. A flowchart for the procurement of urine specimens, processing of biological materials, fusion marker analysis and validation. Urine specimens were procured from 97 PSA‐screened men scheduled for diagnostic biopsy. A total of 92 informative specimens were used for molecular diagnosis, while five specimens were excluded from analyses due to failed RNA extraction or WTA amplification. The dashed line indicates independent confirmation of fusion positive specimens using a second aliquot of original RNA from all 32 fusion‐positive cases. **Figure S2**. A diagram to show the index gene isoforms and the locations of PCR primers used to amplify fusion subtypes. Forward (F) chimeric primers from *TMPRSS2*‐exon 1 were paired with reversed (R) primers from *ERG*‐exons 4, 2, and 5 to amplify *TMPRSS2:ERG* subtypes I, III, and IV. The forward primer from *TMPRSS2*‐exon 1 (TMP‐e1F) was paired with reverse primers from *ETV1* (ETV1‐e6R), *ETV4* (ETV4‐e5R), and *ETV5* (ETV5‐e2R) to amplify corresponding *TMPRSS2:ETS* fusion genes. The fusion partner genes were drawn in proportion to actual structures. **Figure S3.** Dynamic ranges of *TMPRSS2:ERG* subtype markers in serial dilution experiments. Three DNA fragments containing *TMPRSS2:ERG* subtype I, III, and IV were purified and used to generate 10× serial dilutions ranging from 1.8 million to 18 copies for each DNA fragment. Each fusion‐subtype marker was used to amplify the serial dilutions plus a reference urine sample (indicated by red dots and arrows) and a negative control containing no fusion DNA using the protocols described in the Materials and Methods. The qPCR standard curve was constructed for fusion subtypes I (A), III (B) and IV (C) by the MyIQ System Software (v 1.0.410). The *C*_t_ (threshold cycle) was plotted on the *y*‐axis and the log_10_ transformation of the starting material (copy number) on the *x*‐axis. The standard curve was described as: *C*_t_ value = slope × log_10_ (copy number) + *y*‐intercept. The qPCR efficiencies for subtypes I, III, and IV were 94.9%, 91.4%, and 94.1%, respectively. Consistent amplifications were generated in the large dynamic range for each subtype marker, while no signal was generated in the negative control in a 50‐cycle reaction. **Figure S4.** The junction sequences of *TMPRSS2:ERG* subtypes (I, III, and IV) and *TMPRSS2:ETS* fusion genes (*ETV1*, *ETV4* and *ETV5*) validated by DNA sequencing. The arrows indicate the fusion transcript junctions between *TMPRSS2* and *ERG* (NM_004449.3), *ETV1* (NM_004956.3), *ETV4* (NM_001986.1), or *ETV5* (NM_004454.1). The sequence traces were generated using Applied Biosystems 3730xl DNA Analyzer at the McGill University Genome Centre. **Table S1.** Primer sequences of *TMPRSS2:ETS* fusion markers and additional molecular markers. **Table S2.** Combination of fusion markers (or fusion‐types) and raw *C*_t_ values of each fusion marker in urine of 32 fusion‐positive men. **Table S3.** Confirmation of fusion‐positive urine specimens in three corresponding prostatectomy cancer tissues. **Table S4.** Risk groups to cancer occurrence stratified by the FPP molecular scores.
