# Supplementary material for: Early detection of clinically significant prostate cancer at diagnosis: a prospective study using a novel panel of TMPRSS2:ETS fusion gene markers
Source: Cancer Med. 2013 Feb 3;2(1):63–75. doi: 10.1002/cam4.49 (PMC3797559; doi:10.1002/cam4.49)
Supplement: Figure S1. — A flowchart for the procurement of urine specimens, processing of biological materials, fusion marker analysis and validation. Urine specimens were procured from 97 PSA‐screened men scheduled for diagnostic biopsy. A total of 92 informative specimens were used for molecular diagnosis, while five specimens were excluded from analyses due to failed RNA extraction or WTA amplification. The dashed line indicates independent confirmation of fusion positive specimens using a second aliquot of original RNA from all 32 fusion‐positive cases. Figure S2. A diagram to show the index gene isoforms and the locations of PCR primers used to amplify fusion subtypes. Forward (F) chimeric primers from TMPRSS2‐exon 1 were paired with reversed (R) primers from ERG‐exons 4, 2, and 5 to amplify TMPRSS2:ERG subtypes I, III, and IV. The forward primer from TMPRSS2‐exon 1 (TMP‐e1F) was paired with reverse primers from ETV1 (ETV1‐e6R), ETV4 (ETV4‐e5R), and ETV5 (ETV5‐e2R) to amplify corresponding TMPRSS2:ETS fusion genes. The fusion partner genes were drawn in proportion to actual structures. Figure S3. Dynamic ranges of TMPRSS2:ERG subtype markers in serial dilution experiments. Three DNA fragments containing TMPRSS2:ERG subtype I, III, and IV were purified and used to generate 10× serial dilutions ranging from 1.8 million to 18 copies for each DNA fragment. Each fusion‐subtype marker was used to amplify the serial dilutions plus a reference urine sample (indicated by red dots and arrows) and a negative control containing no fusion DNA using the protocols described in the Materials and Methods. The qPCR standard curve was constructed for fusion subtypes I (A), III (B) and IV (C) by the MyIQ System Software (v 1.0.410). The Ct (threshold cycle) was plotted on the y‐axis and the log10 transformation of the starting material (copy number) on the x‐axis. The standard curve was described as: Ct value = slope × log10 (copy number) + y‐intercept. The qPCR efficiencies for subtypes I, III, and IV w [file cam0002-0063-SD1.doc]

**SUPPLEMENTARY TABLES AND FIGURES**

**Suppl. Table S1.** Primer sequences of *TMPRSS2:ETS* fusion markers and additional molecular markers

| Gene^a^ | Primer | Sequence | Amplicon (bp) | Annealing Tm^b^ (^o^C) |
| --- | --- | --- | --- | --- |
| TMP-e1:ERG-e4^c^; subtype I | TMP-e1:ERG-e4F | CTGGAGCGCGGCAGGAA | 65 | 70 |
|  | ERG-e4R | GTAGGCACACTCAAACAACGACTGG |  |  |
| TMP-e1:ERG-e2; subtype III | TMP-e1:ERG-e2F | TGGAGCGCGGCAGGTTATT | 70 | 70 |
|  | ERG-e2R | TTGTCTTGCTTTTGGTCAACACG |  |  |
| TMP-e1:ERG-e5; subtype IV | TMP-e1:ERG-e5F | GGAGCGCGGCAGGAACT | 85 | 70 |
|  | ERG-e5R | GTTCATCCCAACGGTGTCTGG |  |  |
| TMP-e1:ETV1-e6 | TMP-e1F | GGAGCGCCGCCTGGAG | 47 | 70 |
|  | ETV1-e6R | AGTGGCAGGCCATGAAAAGC |  |  |
| TMP-e1:ETV4-e5 | TMP-e1F | GGAGCGCCGCCTGGAG | 68 | 70 |
|  | ETV4-e5R | TGGAAATCAGGAACAAACTGCTCA |  |  |
| TMP-e1:ETV5-e2 | TMP-e1F | GGAGCGCCGCCTGGAG | 104 | 70 |
|  | ETV5-e2R | TCCATGGTGCTTTCAGCGTCT |  |  |
| *PSA* | PSA-e2_3F | GCCCACTGCATCAGGAACAAA | 75 | 70 |
|  | PSA-e3R | CTGGCCTGTGTCTTCAGGATGA |  |  |
| *PCA3* | PCA3-e2:e3F | GGAAGGACCTGATGATACAGAGGTGA | 72 | 70 |
|  | PCA3-e3R | CAGATGTGTGGCCTCAGATGGTA |  |  |
| *ERG(5-6)^d^* | ERG5-6F | CGCAGAGTTATCGTGCCAGCAGAT | 86 | 67 |
|  | ERG5-6R | CCATATTCTTTCACCGCCCACTCC |  |  |
| *ERG(6-7)^d^* | ERG6-7F | AGCTACAACGCCGACATCC | 71 | 67 |
|  | ERG6-7R | GAAGTCAAATGTGGAAGAGGAGTC |  |  |
| *GAPDH* | GAPDH-F | AAGGTCGGAGTCAACGGATTT | 66 | 69 |
|  | GAPDH-R | ACCAGAGTTAAAAGCAGCCCTG |  |  |
| ^a^ *ETV1* ( NM_004956.3), *ETV4* (NM_001986.1), *ETV5* (NM_004454.1), *ERG* (NM_004449.3)  ^b^ The qPCR program consisted of initial denaturing at 95^o^C for 1.5 min, followed by 50 cycles of a two-step reaction at 95^o^C for 15 s and 67-70 ^o^C (varying for marker pairs) for 30 s. The qPCR was performed using the MyiQ real-time PCR system (Bio-Rad).  ^c^ TMP-e1:ERG-e4 stand for fusion between *TMPRSS2*-exon 1 and *ERG*-exon 4. The same interpretation applies to other fusions  ^d^ ERG(5-6) and ERG(6-7) markers refer to ERG-exons (5-6) and ERG-exons (6-7). | | | | |

**Suppl. Table S2.** Combination of fusion markers (or fusion-types) and raw Ct values of each fusion marker in urine of 32 fusion-positive men

| **Samples** | **Cancer status**  **(Gleason score)** | **Fusion-type** | **Ct value (qPCR cycles)^a^** | | | | | |
| --- | --- | --- | --- | --- | --- | --- | --- | --- |
|  |  |  | **TMP:ERG I** | **TMP:ERG III** | **TMP:ERG IV** | **TMP:ETV1** | **TMP:ETV4** | **TMP:ETV5** |
| A124 | 4+3 | TMP:ERG (I + IV) | 35.63 | - | 38.05 | - | - | - |
| A128 | 5+4 | TMP:ERG IV | - | - | 38.03 | - | - | - |
| A138 | 3+4 | TMP:ERG (I + IV) + TMP:ETV1 | 34.81 | - | 37.80 | 30.68 | - | - |
| A161 | 4+3 | TMP:ERG (I + III + IV) | 31.98 | 37.62 | 32.86 | - | - | - |
| A169 | 4+3 | TMP:ERG (I + III) | 32.12 | 38.87 | - | - | - | - |
| A174 | 3+3 | TMP:ERG (I + III) | 32.28 | 34.51 | - | - | - | - |
| A180 | 3+3 | TMP:ERG (I + III + IV) | 29.97 | 35.19 | 36.80 | - | - | - |
| A185 | 5+4 | TMP:ERG (I + IV) | 22.81 | - | 28.26 | - | - | - |
| A197 | 3+3 | TMP:ERG (III) | - | 37.92 | - | - | - | - |
| A198 | 4+4 | TMP:ERG (I + IV) | 21.70 | - | 27.92 | - | - | - |
| A206 | 3+4 | TMP:ETV5 | - | - | - | - | - | 30.87 |
| A209 | 3+3 | TMP:ERG (I + IV) | 27.46 | - | 30.90 | - | - | - |
| A211 | 3+3 | TMP:ERG I | 29.96 | - | - | - | - | - |
| A212 | 3+3 | TMP:ERG (I + III + IV) | 31.10 | 38.59 | 35.68 | - | - | - |
| A213 | 3+4 | TMP:ERG (I + III) | 36.66 | 36.21 | - | - | - | - |
| A224 | 3+3 | TMP:ERG (I + III + IV) | 31.44 | 39.39 | 35.57 | - | - | - |
| A227 | 3+3 | TMP:ETV4 | - | - | - | - | 34.72 | - |
| A228 | 3+3 | TMP:ERG (I + III) | 33.71 | 35.62 | - | - | - | - |
| A229 | 3+3 | TMP:ERG (I + III + IV) | 31.09 | 37.12 | 32.33 | - | - | - |
| A230 | 3+3 | TMP:ERG (I + III) | 35.13 | 38.11 | - | - | - | - |
| A235 | 3+4 | TMP:ERG (I + III) | 33.30 | 36.72 | - | - | - | - |
| A120 | benign | TMP:ERG I | 34.16 | - | - | - | - | - |
| A168 | benign | TMP:ERG (I + III + IV) | 35.34 | 38.30 | 34.31 | - | - | - |
| A175 | benign | TMP:ERG III | - | 38.50 | - | - | - | - |
| A187 | benign | TMP:ERG (I + III) | 36.38 | 39.57 | - | - | - | - |
| A188 | benign | TMP:ERG (I + III + IV) | 32.72 | 34.13 | 35.10 | - | - | - |
| A195 | benign | TMP:ERG I + TMP:ETV5 | 34.03 | - | - | - | - | 23.07 |
| A203 | benign | TMP:ERG I | 34.85 | - | - | - | - | - |
| A215 | benign | TMP:ERG I | 33.15 | - | - | - | - | - |
| A217 | benign | TMP:ERG I | 34.70 | - | - | - | - | - |
| A218 | benign | TMP:ERG I | 35.80 | - | - | - | - | - |
| A222 | benign | TMP:ERG I | 31.99 | - | - | - | - | - |
| ^a^ The qPCR program consisted of initial denaturing at 95^o^C for 1.5 min, followed by 50 cycles of a two-step reaction at 95^o^C for 15 s and 64-70 ^o^C (varying for each marker) for 30 s. The qPCR was performed using the MyiQ real-time PCR system (Bio-Rad). | | | | | | | | |

**Suppl. Table S3.** Confirmation of fusion-positive urine specimens in three corresponding prostatectomy cancer tissues ^a^

|  | Urine sample (ΔΔCT) | | | | | |  | Prostatectomy tissue (ΔΔCT) | | | | | |
| --- | --- | --- | --- | --- | --- | --- | --- | --- | --- | --- | --- | --- | --- |
| samples | PSA^b^ | PCA3 | ERG5-6 | TMP:ERG I^c^ | TMP:ERG III | TMP:ERG IV |  | PSA^b^ | PCA3 | ERG5-6 | TMP:ERG I^c^ | TMP:ERG III | TMP:ERG IV |
| A161 | 81.01 | 117.78 | 751.35 | 3.77 | 13.99 | 3.64 |  | 1516.64 | 490.01 | 4629.58 | 115.36 | 0.07 | 3.96 |
| A213 | 26.60 | 193.12 | 149.43 | 0.05 | 5.67 | NQ |  | 1482.00 | 2765.52 | 5281.26 | NQ | 61714.99 | NQ |
| A230 | 27.54 | 66.72 | 26.29 | 0.29 | 17.55 | NQ |  | 3162.10 | 744.43 | 352.95 | NQ | 321.05 | NQ |
| ^a^ Leftover paraffin-embedded prostatectomy cancer tissues from the corresponding pre-biopsy urine samples were used for RNA extraction and whole transcriptome (WTA) amplification; both fusion and non-fusion markers were analyzed using the same protocol as for the urine specimens.  ^b^ Non-fusion markers (urine *PSA*, *PCA3* and *ERG5-6*) were normalized to *GAPDH* and quantified using a common calibrator urine sample A092.  ^c^ Fusion markers (*TMPRSS2:ERG* subtypes I, III and IV) were normalized to *GAPDH* and quantified using a calibrator urine sample A229 that was fusion positive. | | | | | | | | | | | | | |

**Suppl. Table S4.** Risk groups to cancer occurrence stratified by the FPP molecular scores.

(Note: unlike the Table 4B where the relative risk to clinically significant features was calculated only in 39 biopsy-positive cases, all 92 pre-biopsy cases were used in relative risk analysis in this supplementary table. The significantly increased relative risks to clinically significant features in this table were due to over-optimization in data analysis when biopsy-negative cases were included)

|  | High | Intermediate | Low |
| --- | --- | --- | --- |
| Risk score^a^ |  |  |  |
| n (%) | 31 (34%) | 30 (33%) | 31 (34%) |
| median (min-max) | 0.7967 (0.4707-0.9391) | 0.3077 (0.2415-0.4582) | 0.1772 (0.0943-0.2335) |
| Cancer |  |  |  |
| n (%)^b^ | 25 (81%) | 9 (30%) | 5 (16%) |
| RR (H *vs.* L), *p* ^c^ | 5.000 (2.199-11.370), *p*<0.0001 | | |
| RR (H *vs.* (I+L)), *p* ^d^ | 3.514 (2.150-5.743), *p*<0.0001 | | |
| Gleason score ≥ 7 |  |  |  |
| n (%)^b^ | 14 (45%) | 3 (10%) | 0 (0%) |
| RR (H *vs.* L), *p* | undefined, *p*<0.0001 | | |
| RR (H *vs.* (I+L)), *p* | 9.183 (2.850-29.580), *p*<0.0001 | | |
| High risk of recurrence by NCCN^e^ | |  |  |
| n (%)^b^ | 7 (28%) | 2 (7%) | 0 (0%) |
| RR (H *vs.* L), *p* | undefined, *p*=0.0108 | | |
| RR (H *vs.* (I+L)), *p* | 6.672 (1.470-30.280), *p*=0.0071 | | |
| # of cores positive ≥ 3 |  |  |  |
| n (%)^b^ | 16 (52%) | 6 (20%) | 2 (6%) |
| RR (H *vs.* L), *p* | 8.000 (2.006-31.910), *p*=0.0002 | | |
| RR (H *vs.* (I+L)), *p* | 3.935 (1.896-8.171), *p*=0.0001 | | |
| Max. % cancer inv. in any core ≥ 50% | |  |  |
| n (%)^b^ | 12 (39%) | 2 (7%) | 0 (0%) |
| RR (H *vs.* L), *p* | undefined, *p*=0.0001 | | |
| RR (H *vs.* (I+L)), *p* | 6.000 (1.462-24.63), *p*=0.0050 | | |
| ^a^ FPP molecular score: Fx (III, IV, ETS)+*PCA3*+pre-Bx PSA score  ^b^% of each risk group or positive predictive value in a total of 92 pre-biopsy patients  ^c^ Relative risk between high (H) and low (L) risk groups with 95% CI, p value of Fisher exact test  ^d^ Relative risk between high and intermediate + low (I + L) risk groups with 95% CI, p value of Fisher exact test  ^e^ Gleason score ≥ 8, PSA ≥ 20 ng/ml or T3a as defined by the NCCN guidelines | | | |


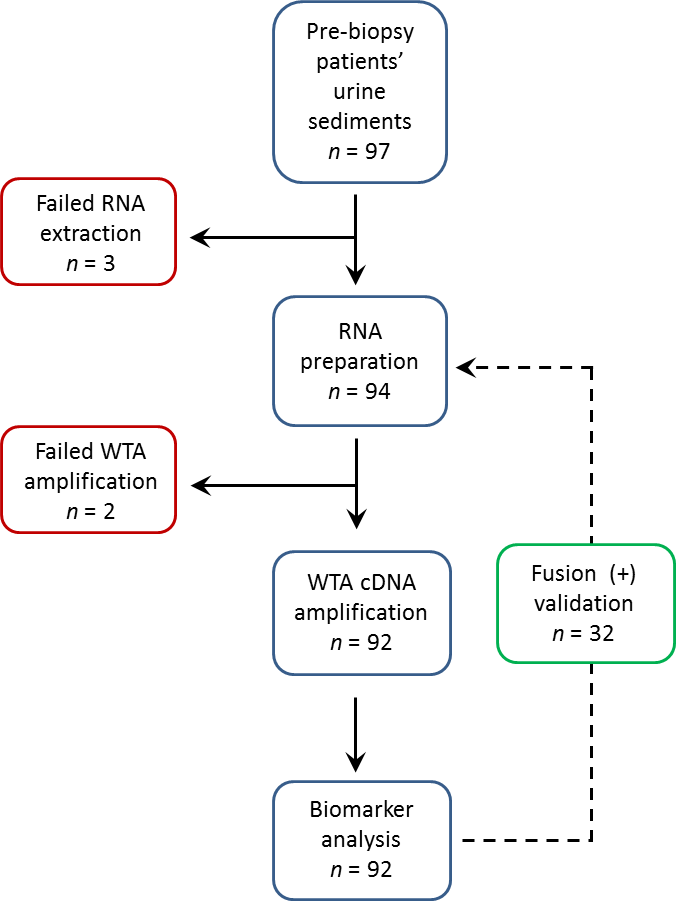


**Suppl. Figure S1.** A flow-chart for the procurement of urine specimens, processing of biological materials, fusion marker analysis and validation. Urine specimens were procured from 97 PSA-screened men scheduled for diagnostic biopsy. A total of 92 informative specimens were used for molecular diagnosis, while 5 specimens were excluded from analyses due to failed RNA extraction or WTA amplification. The dashed line indicates independent confirmation of fusion positive specimens using a second aliquot of original RNA from all 32 fusion-positive cases.


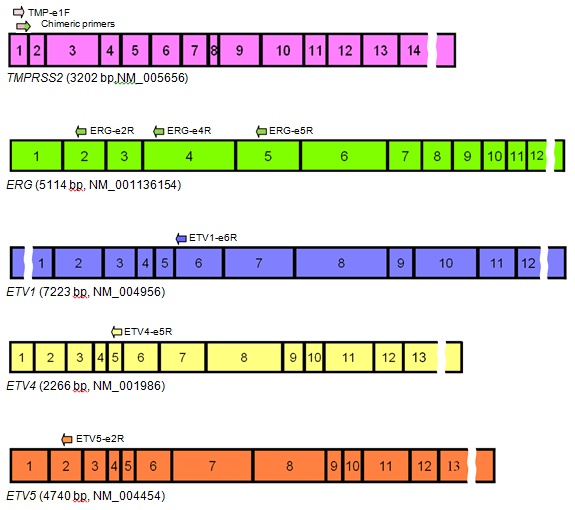


**Suppl. Figure S2.** A diagram to show the index gene isoforms and the locations of PCR primers used to amplify fusion subtypes. Forward (F) chimeric primers from *TMPRSS2*-exon 1 were paired with reversed (R) primers from *ERG*-exons 4, 2 and 5 to amplify *TMPRSS2:ERG* subtypes I, III and IV. The forward primer from *TMPRSS2*-exon 1 (TMP-e1F) was paired with reverse primers from *ETV1* (ETV1-e6R), *ETV4* (ETV4-e5R) and *ETV5* (ETV5-e2R) to amplify corresponding TMPRSS2:ETS fusion genes. The fusion partner genes were drawn in proportion to actual structures.

TMP:ERG I (TMP-e1:ERG-e4)


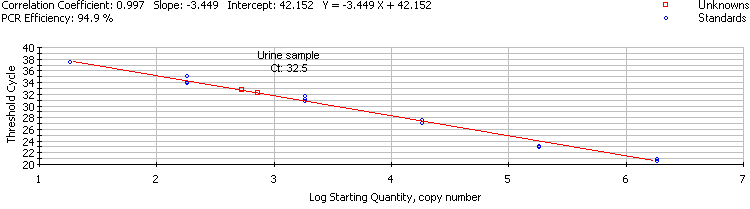


TMP:ERG III (TMP-e1:ERG-e2)


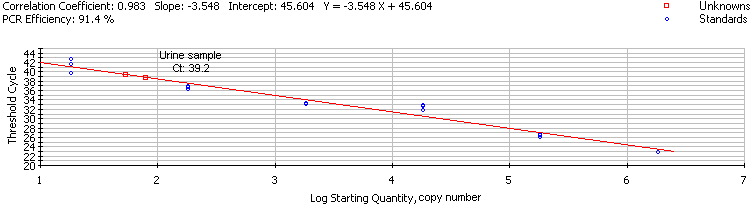


TMP:ERG IV (TMP-e1:ERG-e5)


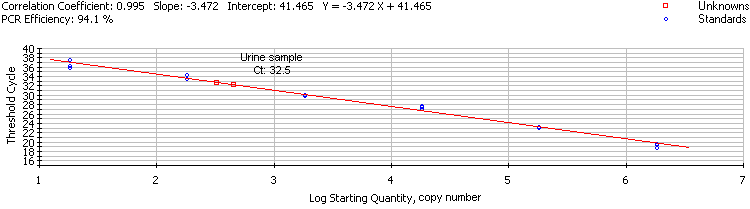


**Suppl. Figure S3.** Dynamic ranges of *TMPRSS2:ERG* subtype markers in serial dilution experiments. Three DNA fragments containing *TMPRSS2:ERG* subtype I, III and IV were purified and used to generate 10 x serial dilutions ranging from 1.8 million to 18 copies for each DNA fragment. Each fusion subtype marker was used to amplify the serial dilutions plus a reference urine sample (indicated by red dots and arrows) and a negative control containing no fusion DNA using the protocols described in the Materials and Methods. The qPCR standard curve was constructed for fusion subtypes I (A), III (B) and IV (C) by the MyIQ System Software (v 1.0.410). The Ct (threshold cycle) was plotted on the y-axis and the log10 transformation of the starting material (copy number) on the x-axis. The standard curve was described as: Ct value = slope x log_10_ (copy number) + y-intercept. The qPCR efficiencies for subtypes I, III and IV were 94.9%, 91.4% and 94.1%, respectively. Consistent amplifications were generated in the large dynamic range for each subtype marker, while no signal was generated in the negative control in a 50-cycle reaction.


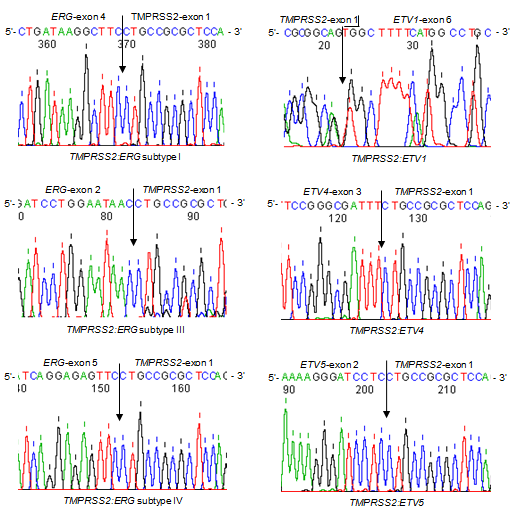


**Suppl. Figure S4.** The junction sequences of *TMPRSS2:ERG* subtypes (I, III and IV) and *TMPRSS2:ETS* fusion genes (*ETV1*, *ETV4* and *ETV5*) validated by DNA sequencing. The arrows indicate the fusion transcript junctions between *TMPRSS2* and *ERG* (NM_004449.3), *ETV1* (NM_004956.3), *ETV4* (NM_001986.1) or *ETV5* (NM_004454.1). The sequence traces were generated using Applied Biosystems 3730xl DNA Analyzer at the McGill University Genome Centre.
